# New Composite Materials Based on PVA, PVP, CS, and PDA

**DOI:** 10.3390/polym16233353

**Published:** 2024-11-29

**Authors:** Muhammad Tahir, Silvia Vicini, Tomasz Jędrzejewski, Sylwia Wrotek, Alina Sionkowska

**Affiliations:** 1 Department of Biomaterials and Cosmetic Chemistry, Faculty of Chemistry, Nicolaus Copernicus University, Gagarina 7, 87-100 Torun, Poland; 2 Department of Chemistry and Industrial Chemistry, University of Genova, 16146 Genoa, Italy; silvia.vicini@unige.it; 3 Department of Immunology, Faculty of Biological and Veterinary Sciences, Nicolaus Copernicus University, Lwowska 1, 87-100 Torun, Poland; tomaszj@umk.pl (T.J.); wrotek@umk.pl (S.W.)

**Keywords:** polymers, composites, polydopamine, polyvinyl alcohol, chitosan, polyvinyl pyrrolidone, polymeric composites, wound healing

## Abstract

In this work, new materials based on the blends of polyvinyl alcohol (PVA), polyvinyl pyrrolidone (PVP), chitosan (CS), and polydopamine (PDA) have been prepared. Fourier Transform Infrared Spectra have been conducted to verify the presence of individual components in the composite materials. EDX elemental analysis showed a clear view of the element’s presence in the composite materials, with the maximum values for carbon and oxygen. Atomic force microscopy (AFM) was used to observe the surface topography and measure the surface roughness. In the case of the individual polymers, CS presented the higher value of surface roughness (Rq = 3.92 nm and Ra = 3.02 nm), and surface roughness was found to be the lowest in the case of polyvinyl pyrrolidone (PVP), and it was with values (Rq = 2.34 nm and Ra = 0.95 nm). PVA films presented the surface roughness, which was with the value (Rq = 3.38 nm and Ra = 2.11 nm). In the case of composites, surface roughness was highest for the composite based on PVA, PVP, and CS, which presented the value (Rq = 11.91 nm and Ra = 8.71 nm). After the addition of polydopamine to the polymeric composite of PVA, PVP, and CS, a reduction in the surface roughness was observed (Rq = 7.49 nm and Ra = 5.15 nm). The surface roughness for composite materials was higher than that of the individual polymers. The addition of PDA to polymeric composite (PVA/PVP/CS) led to a decrease in Young’s modulus. The elongation percentage of the polymeric films based on the PVA/PVP/CS/PDA blend was higher than that of the blend without PDA (9.80% vs. 5.68% for the polymeric composite PVA/PVP/CS). The surface of polymeric films was hydrophilic. The results from the MTT assay showed that all tested specimens are non-toxic, and it was manifested by a significant increase in the viability of L929 cells compared with control cells. However, additional studies are required to check the biocompatibility of tested samples.

## 1. Introduction

In the last three decades, an increasing interest in new materials based on blends of two or more polymers has been observed. Polymer blends may offer new interesting properties, such as improved mechanical properties and biocompatibility, compared with those of single components [1]. Several polymers have been considered in medical applications as wound healing materials [2]. Polyvinyl alcohol (PVA) is prominently vital in wound healing and tissue engineering; Thai et al. [3] highlighted that the main reason for polyvinyl alcohol’s application in wound healing is its biocompatibility. Alghasham [4] and Alipour et al. [5] also stress the biocompatibility for the medical applications of PVA. Polyvinyl alcohol is well known for its attractive properties, such as high surface area and hydrophilic nature [6]. PVA has a strong ability to adhere and numerous contributions to the environment [7]. Safety is also an important feature to be noticed while pharmaceutical applications are concerned. Mohite et al. [8] found PVA safe for biomedical applications. Sharma et al. [9] reported the absence of carcinogenicity in the case of PVA. Narayanan et al. [10] showed that PVA holds good mechanical properties for application in wound healing dressing. Babae et al. [11] conducted a study about the loading of PVA with active substances, and it has been concluded that PVA-loaded materials have the ability to boost the wound healing process. Soleiman-Dehkordi et al. [12] mentioned that good mechanical properties of PVA ensure suitability for tissue engineering applications. Huang et al. [13] obtained the hydrogel composed of chitosan (CS) and PVA, and it has been observed to reduce inflammation around the cells. Shankhwar et al. [14] developed a product comprising PVA and polyvinyl pyrrolidone (PVP), and it has been concluded that the prepared composite presented excellent cell proliferation results and provided an antimicrobial barrier. So, it has been suggested that the blend of the above-mentioned polymers can be considered during wound dressing preparation.

Natural polymers can be used in wound healing applications as bioactive materials, which may enhance regeneration. One of the most commonly used biopolymers in wound healing is chitosan. Chitosan is usually obtained by following the deacetylation route of chitin [15]. Elizalde-Cárdenas et al. [16] discussed the reason for the deacetylation of the chitin to chitosan, which is the easy modification of the chitosan. Wang et al. [17] put detailed insight into the modification of chitosan, and notable modifications of chitosan were chemical, physical, and enzymatic modifications. It has been emphasized by Torkaman et al. [18] that for wound healing applications, chitosan modification is possible by blending, as it has been mentioned as part of the physical modification of chitosan. Polymeric blending of synthetic and natural polymers is key in improving biocompatibility and mechanical properties. Numerous applications of polymeric blends based on chitosan for biomedical applications have been reported [1]. Shokri et al. [19] well drafted the enzymatic modification of chitosan, and it has been reported that enzymatic modification is primarily responsible for the modification of the physicochemical properties. Alves et al. [20] described the enzymatic modification of chitosan derivatives that enhanced hydrophilicity and adhesion. Lunkov et al. [21] focused on the chemical modification of chitosan and described that hemostatic properties enhancement is possible by chemical modification of chitosan. Tang et al. [22] also briefed about the hemostatic behavior of chitosan, whereas Li et al. [23] mentioned the bleeding control by chitosan in the case of the wound healing. The skin wound healing process was promoted by limiting the excessive rate of inflammation, and this limitation of excessive inflammation was possible by the transformation of the M1 macrophages to M2, as discussed in this study [24]. Huang et al. [25] wrote about this transformation and highlighted that the limitation of the inflammation led to the increment of the vascular endothelial growth factor (VEGF). This increment of the VEGF led to the promotion of wound healing. Zhang et al. [26] presented a well-described role of the chitosan-based hydrogels in transforming the macrophages to the M2 type and also mentioned skin wound healing in this study. The role of chitosan in enhancing the proliferative biomarker content was noted in the study conducted by Le et al. [27]. Chitosan has applications in four stages of wound healing, namely hemostasis, inflammation, proliferation, and remodeling, as indicated by Rajinikanth B et al. [28]. Ara et al. described that chitosan-based hydrogels have angiogenic potential that helps the early wound healing process [29]. Zhao et al. reported the anti-bacterial effect of the chitosan [30]. Sanmugam et al. reported that the anti-bacterial properties of chitosan play a beneficial role in wound healing [31]. Chou et al. reported that chitosan presented platelet adhesion and activation, and it was the study based on the rabbit model [32]. Kordestani et al. [33] demonstrated that chitosan hydrogel led to early scar tissue formation, and scar tissue has a prominent role in the remodeling phase of wound healing. Chitosan can be modified by blending it with another polymer, for example, polyvinyl pyrrolidone (PVP). A detailed study about blends of chitosan and PVP was published by Lewandowska [34]. 

PVP is a synthetic polymer, and its solubility is highly dependent on the polarity of the solvent [35,36,37]. Due to its film-forming properties and biocompatibility, PVP is widely used in the biomedical field. Zheng et al. [38] have discussed the application of PVP in wound dressing, as it fulfills the core requirement of biocompatibility. Solanki et al. [39] also highlighted applications of PVP in biomedicine and pharmaceuticals. Gounden and Singh [36] have mentioned that PVP has a beneficial role in debridement, and the debridement process is defined by Mayer et al. [40] as eliminating non-viable or dead tissues. Shoham et al. [41] reported debridement as the exposure to the wound bed and highlighted the importance of the debridement process for the viability of the dermis. PVP has the tendency to absorb the extra wound exudate, which is the major reason for its use in the debridement process [42]. Contardi et al. [43] showed that PVP is hemocompatible and useful for wound dressing applications. Razzak et al. [44] prepared the hydrogel based on PVA and PVP for the wound dressing applications, and the prepared hydrogel effectively absorbed the fluid. Raza et al. [45] discussed several fabrication techniques of hydrogels based on PVP and CS for wound dressing. 

In our research, we prepared a blend of PVA, PVP, and CS. Polydopamine (PDA) has been added to modify the properties of a blend. PDA can be prepared by polymerizing dopamine [46]. Milyaeva et al. discussed the dark brown color of polydopamine [47]. Liu et al. prepared the polydopamine nanoparticles; the reported color of polydopamine nanoparticles in the study was dark brown [48]. Li et al. highlighted the dark black color of polydopamine in its solid form [49]. Ferrer et al. made a detailed discussion about the color of polydopamine in solid form and reported that the color in their study was dark black [50]. PDA has good adhesive properties, and its biocompatibility has been provenly discussed in the case of in vivo and in vitro, as it has applications in wound healing [51]. Polydopamine was found to be biodegradable and well considered due to its surface properties [52,53]. PDA is also used in biomedical applications due to its hydrophilic characteristics [54,55]. Hu et al. [56] also stated the anti-oxidative properties of PDA. The applications of PDA in wound healing have been summarized in their research as well. PDA has a strong ability for free radical scavenging and may play an influential role as an anti-bacterial agent. PDA was already used as an additive to polymers [57]. Wang et al. [58] prepared a hydrogel consisting of PVA and PDA. Zheng et al. [59] prepared a hydrogel based on polydopamine and polyvinyl alcohol in combination with the tea phenols, and it has been analyzed to be fit for the preparation of wound dressing because of its cytocompatibility.

The selection of components for the fabrication of complex composite materials is based on the collective properties of PVA, CS, PVP, and PDA. PVA is biocompatible, hydrophilic, and has a strong ability to adhere. So, it is safe to use in biomedical applications. Chitosan has attractive applications in four stages of wound healing. Chitosan has a natural origin, which also enhances its biomedical applications. The prominent role of chitosan is in the hemostasis stage of wound healing to control the bleeding. The ease of modification also supports chitosan applications in the biomedical field. PVP shows hemocompatibility, which is its best advocate for use in the biomedical field in the form of wound dressings. Polydopamine found applications in the biomedical field due to its unique properties like cytocompatibility, anti-oxidative, and anti-bacterial properties. The adhesive nature of polydopamine makes it perfectly suitable as a bio-adhesive in the biomedical field. So, we can hypothesize that composite materials based on PVA, PVP, CS, and PDA will present the unique properties of each component for the wound healing applications. 

In this research, we have prepared a set of composites based on PVA, PVP, CS, and PDA. After the extensive literature study, we believe this composite type has not been analyzed until today. So, a preliminary study is needed to give insight into such composites based on polymer blends and their potential for biomedical applications.

## 2. Materials and Methods

Dopamine hydrochloride (CAS-No.:62-31-7), chitosan (high molecular weight, 102537320, CAS-No:9012-76-4), polyvinyl alcohol (medium molecular weight (M.W: 30,000–70,000 Da), 102649077, CAS-No.:9002-89-5, 87–90% degree of hydrolysis), polyvinyl pyrrolidone (M.W: 40,000 Da, 102652840, CAS-No.:9003-39-8) obtained from Sigma Aldrich, Darmstadt, Germany. Aqueous ammonia (CAS-No.:1336-21-6, 25%) has been utilized (POCH SA, Gliwice, Poland). Acetic acid (CAS:64-19-7, 99.9%) was obtained from STANLAB, Lublin, Poland. 

### 2.1. Preparation of Polymeric Solutions

Polymeric solutions of PVA, PVP, and CS have been prepared. PVA and PVP have been dissolved in distilled water at a concentration of 5% (wt/v). The polymeric solution of CS was prepared in the aqueous acetic acid solution of 0.1 M, and the concentration of the chitosan solution was kept at 1% (wt/v).

### 2.2. Polydopamine Preparation

Dopamine hydrochloride has been converted to polydopamine. The conversion of dopamine hydrochloride to polydopamine was essential to get PDA and also because dopamine hydrochloride has a toxic nature, as mentioned in the safety data sheet (SDS). In contrast, polydopamine is non-toxic and widely used for biomedical applications. The reaction mechanism has been followed, as mentioned by Gao et al. [60]. Firstly, the dopamine hydrochloride (50 mg) was taken and put into 1 mL of distilled water. Then, dopamine hydrochloride, already dissolved in distilled water, was blended with 9 mL of distilled water, 5 mL of ethanol, and 0.15 mL of the aqueous ammonia in the flask. The color change was evident as the mixing process occurred, initially leading to the pale-yellow color. Then, with time, as the oxidation and polymerization process progressed, the color started turning black from the pale-yellow color. After 24 h, the color observed was dark brown. PDA was obtained following the reaction mechanism of Gao et al. [60], and we utilized polydopamine (5% wt.) for the composite material. This amount of PDA was used because we observed that the stable polymeric film can be fabricated. 

### 2.3. Preparation of Composite Material of PVA, PVP, CS, and PDA

The composite of polyvinyl alcohol, chitosan, and polyvinyl pyrrolidone has been initially prepared. The polymeric blend of the composite was prepared by mixing in the proportion of 40:10:50 for PVA, PVP, and CS, respectively, at room temperature. The polymeric blend has been prepared by mixing CS, PVA, and PVP with dark brown supernatant PDA at room temperature in the proportion of 50:40:5:5. The solvent casting method has been selected to prepare the polymeric films. In the solvent casting technique, the polymeric solvent is allowed to evaporate, preparing stable polymeric films. The pH of the composite solution of PVA, PVP, CS, and PDA was 4. Acidic pH is needed for dissolution of chitosan. Moreover, Kalaycıoğlu et al. [61] highlighted the acidic pH of the commercial wound dressings. Ferrante et al. [62] discussed the hydrogel composed of chitosan/pectin/NaCl with a pH of 4, which exhibited anti-bacterial properties, and hydrogel has been recommended for potential wound dressing applications. Razack et al. [63] mentioned the advantageous role of slightly acidic pH in promoting fibroblast proliferation and preventing bacterial infection. The composition of the prepared composite materials has been presented in Table 1.

### 2.4. Scanning Electron Microscopy (SEM-EDX)

Polymeric films have been analyzed by scanning electron microscopy (SEM), which has numerous impacts on the surface morphology. The scanning electron microscope (LEO, Electron Microscopy Limited, Cambridge, UK) was used for the analysis in this research. The chemical composition of the individual polymers and polymeric composites has been analyzed by utilizing the Quantax 200 Energy Dispersive X-ray (EDX) spectrometer (Bruker AXS, Germany), and it was equipped with the detector XFlash 4010. Gold coating was used for the SEM analysis. 

### 2.5. Fourier Transform Infrared Spectroscopy (FTIR)

Fourier Transform Infrared Spectroscopy (FTIR) was utilized to analyze the functional groups in the obtained polymeric films. This study used a Nicolet iS10 spectrometer equipped with an ATR device (Thermo Fisher Scientific, Waltham, MA, USA). The parameters were chosen: range 400–4000 cm^−1^, resolutions = 4 cm^−1^, and scans = 64. The observation of the FTIR spectrum was made by Omnic 2009. The FTIR spectrum as a graph was generated using the Origin Pro software (https://www.originlab.com).

### 2.6. Atomic Force Microscopy (AFM)

The surface roughness of the polymeric films was analyzed using atomic force microscopy (AFM). An atomic force microscope (Veeso Digital Instrument, Santa Barbara, CA, USA) has been used to analyze/scan the samples. The selection criteria were based on the standard room temperature, while the atmospheric pressure was maintained for surface roughness analysis with the AFM. The scans have been conducted for the area of 10 µm × 10 µm. The surface roughness was calculated using NanoScope Analysis (Bruker, Ettlingen, Germany). AFM analysis has been carried out at the resonant frequency of 140 KHz and an elastic/spring constant of 3.5 N/m. 

### 2.7. Mechanical Properties

The samples (5 cm × 1 cm) for mechanical properties have been prepared by cutting the polymeric films. The mechanical properties testing machine (Z.05, Zwick and Roell, Ulm, Germany) was applied to evaluate the mechanical properties. The parameters for the measurements of mechanical properties were as follows: initial force was 0.1 MPa, testing speed was 50 mm/min, initial force speed was 5 mm/min, and cell load was 0.5 N. The results of the mechanical properties were viewed and recorded using the testXpert II 2017 program.

The recorded results of the mechanical properties have been imported into the Jupyter Notebook (Python). A summary statistics table of mechanical properties was also generated, and it provided the mean values. The standard error values have been calculated by importing sem from scipy.stats. Firstly, the normality of data was analyzed using the Shapiro–Wilk test by importing the shapiro function from scipy.stats. The *p*-values in the Shapiro–Wilk test for the analyzed data were greater than 0.05, confirming the normal distribution of the data. Once the normal distribution is confirmed, the parametric test, like one-way analysis of variance (ANOVA), is applied by importing f_oneway from scipy.stats library. The value of *p* from the ANOVA test was analyzed lower than 0.05, which clearly indicated the statistical significance of the data. Tukey post hoc test has also been conducted by importing the pairwise_tukeyhsd from the statsmodels.stats.multicomp. The significance value alpha specified for pairwise_tukeyhsd was 0.05. The graphs of the mechanical properties have been generated utilizing Plotly Express in Python.

### 2.8. Contact Angle and Surface Energy

The contact angle measurements have been noted utilizing the goniometer equipped with drop shape analysis (DSA 10, Krüss, Hamburg, Germany). Experiments were conducted at room temperature; glycerin and diiodomethane were used for the contact angle analysis on the polymeric films. The contact angle value is presented in Section 3 “Results and Discussion” as the average value with the standard deviation, and the surface energy calculations have been obtained by drop shape analysis (DSA 10, Krüss, Hamburg, Germany). Nine contact angle measurements have been recorded for each polymeric film with glycerin and diiodomethane. In each measurement, a single drop of the glycerin and diiodomethane has been used. 

### 2.9. Cell Culture and Cytotoxicity Evaluation

Murine fibroblast cell line L929 (European Collection of Authenticated Cell Cultures (Salisbury, UK; Cat. No. 85011425) was cultured in RPMI 1640 medium supplemented with 10% heat-inactivated fetus bovine serum (FBS), 100 µg/mL streptomycin, and 100 IU/mL penicillin. The cells collected between 10 and 15 passages were maintained at 37 °C in a humidified atmosphere with 5% CO_2_ and passaged using 0.25% trypsin-EDTA solution when reaching 70–80% of confluence. All reagents used for cell culture were purchased from VWR International (Radnor, PA, USA).

The indirect cytotoxicity of tested polymers (PVA, CS) and polymeric composites (PVA/PVP/CS, PVA/PVP/CS/PDA) was evaluated using the extraction method based on a procedure adapted from the International Organization for Standardization: standards test method ISO 10993-5:2021 [64] and ISO 10993-12:2021 [65]. Before experiments, for sterilization, all samples were placed under a laminar chamber for UV exposure lasting 30 min for each side. Then, the specimens (0.1 g) were soaked in 4 mL of RPMI 1640 medium, which was completely absorbed by the films, followed by using an extraction ratio of 0.2 g of samples per 1 mL of RPMI 1640 medium. The films were incubated for 24 h at 37 °C in a humidified atmosphere with 5% CO_2_, followed by centrifugation (2000× *g*, 5 min) to remove all debris.

To determine the viability of L929 cells, the MTT assay was used to detect the reduction of MTT (3-(4,5-dimethylthiazolyl)-2,5-diphenyl-tetrazolium bromide; Merck KGaA, Darmstadt, Germany) by mitochondrial dehydrogenase to a formazan, which reflects the normal functioning of mitochondria of live cells. For each experiment, 5 × 10^3^ cells/well were seeded in 96-well plates and pre-incubated for 24 h. Then, L929 cells were stimulated with undiluted extraction medium (1), diluted 1:2 and 1:4 for 24, 48, and 72 h. The control cells were cultured in the corresponding dilution of RPMI 1640 medium, which was prepared similarly to the extracts. Blank wells contained diluted samples or culture control medium without cells. After stimulation, 100 µL/well of MTT solution (0.5 mg/mL of MTT reagent in PBS) was added to each well. Plates were incubated at 37 °C for 3 h. Subsequently, the formazan formed by viable cells was dissolved in 100% dimethyl sulfoxide (50 µL/well). The optical density was measured at 570 nm (with a reference wavelength of 630 nm) using the Synergy HT Multi-Mode Microplate Reader (BioTek Instruments, Winooski, VT, USA). The results were presented as a percentage (mean ± standard deviation) of the control cells, which served as 100%. The GraphPad Prism 7.0 software (GraphPad Software Inc., La Jolla, CA, USA) was used for statistical analyses of the results from the MTT assay. These results were analyzed using a one-way analysis of variance (ANOVA) with the post hoc Tukey test. The level of significance was set at *p* < 0.05. The MTT assays were repeated in three separate experiments.

## 3. Results and Discussion

### 3.1. Fourier Transform Infrared Spectroscopy (FTIR)

**Figure 1:**
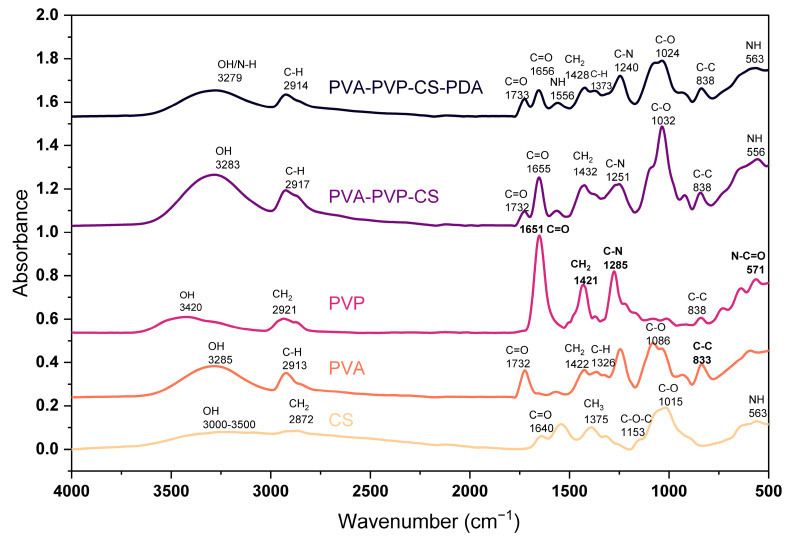
FTIR spectrum of polymers and composite materials.

IR spectra of the polymers and composite materials have been presented in Figure 1. For CS polymeric film, we found major peaks at 563 cm^−1^, 1015 cm^−1^, 1153 cm^−1^, 1375 cm^−1^, 1544 cm^−1^, 1640 cm^−1^, and 2872 cm^−1^, and a wide band in the range of 3000–3500 cm^−1^. The band around 3000–3500 cm^−1^ indicates the O–H group in the polymer structure, as reported in the research conducted by Rubentheren et al. [66]. The peak at 563 cm^−1^ is due to the bending of NH, as highlighted by Varma and Vasudevan [67]. The peak at 1015 cm^−1^ is the effect of C–O stretching vibration of the alcohol groups; similar results have been reported by El-Araby et al. [68]. The peak at 1153 cm^−1^ presents asymmetric stretching of C–O–C, and several studies have confirmed results [69,70]. The CH_3_ deformation peaked at 1375 cm^−1^, as pointed out in the studies [71,72,73]. The peak at 1640 cm^−1^ is the C=O stretching of amide I, as it has been claimed by Gonzaga et al. [74]. The peak at 2872 cm^−1^ resulted from the stretching of CH_2_ in polymer chains; a similar result has been reported in the study by Mourya et al. [75].

We found the major peaks for PVA films at 833 cm^−1^, 1086 cm^−1^, 1326 cm^−1^, 1422 cm^−1^, 1732 cm^−1^, 2913 cm^−1^, and 3285 cm^−1^. The peaks appearing at 833 cm^−1^, 1086 cm^−1^, 1326 cm^−1^, and 1422 cm^−1^ are due to the C–C stretching, C–O stretching, C–H deformation, and CH_2_ bending, respectively, as Kharazmi et al. [76] discussed. The peak observed at 1732 cm^−1^ highlights the C=O stretching, and it is a result of the acetate carbonyl group, while the symmetrical stretching of C–H has been noticed at 2913 cm^−1^. Sankarganesh et al. [77] presented a similar peak analysis. The band at 3285 cm^−1^ is due to the presence of an OH group in the polymer chain.

PVP spectrum shows the major peaks at 571 cm^−1^, 645 cm^−1^, 1285 cm^−1^, 1421 cm^−1^, 1651 cm^−1^, 2921 cm^−1^, and at 3420 cm^−1^. The peak at 571 cm^−1^ highlights N–C=O bonding, which has been confirmed in the study conducted by Machmudah et al. [78]. The peak at 1285 cm^−1^ indicates the C–N stretching, and at 1421 cm^−1^, it shows the CH_2_ bending; similar cases have been reported in the study by Li et al. [79]. The C=O stretching led to the peak at 1651 cm^−1^, as stated by Yao et al. [80]. A peak at 2921 cm^−1^ is the effect of the symmetrical vibration of the CH_2_, as mentioned by Rahma et al. [81]. Alshammari et al. [82] clearly described the peak at 3420 cm^−1^ as the vibrational stretch of the OH (hydroxyl) group.

The composite based on PVA, PVP, and CS presented the peaks at 556 cm^−1^, 838 cm^−1^, 1032 cm^−1^, 1251 cm^−1^,1655 cm^−1^, 2917 cm^−1^, and 3283 cm^−1^. The peak at 556 cm^−1^ in the composite indicates NH bending, which ensures the presence of CS. The peak at 1655 cm^−1^ is the presentation of the C=O stretching, which indicates the PVP. The peak at 2917 cm^−1^ is due to the symmetrical C–H stretching, which gives signals of PVA availability. The composite material consisting of PVA, PVP, CS, and PDA presented the major peaks at 563 cm^−1^, 838 cm^−1^, 1024 cm^−1^, 1240 cm^−1^, 1373 cm^−1^, 1428 cm^−1^, 1556 cm^−1^, 1656 cm^−1^, 1733 cm^−1^, 2914 cm^−1^, and 3279 cm^−1^. It has been checked in the literature, and polydopamine peaks at 1629 cm^−1^, 1554 cm^−1^, 1518 cm^−1^, 1294 cm^−1^, and 1035 cm^−1^ have been mentioned by Ruppel and Liang [83], while polydopamine peaks reported by Rahoui et al. [84] at 1288 cm^−1^, and 1382 cm^−1^ are the identification of the polydopamine presence due to the CH_2_ and OH catechol groups of polydopamine. So, there is significant evidence of polydopamine peaks when we compare our results to those reported in the literature studies. 

### 3.2. Scanning Electron Microscopy (SEM)

**Figure 2:**
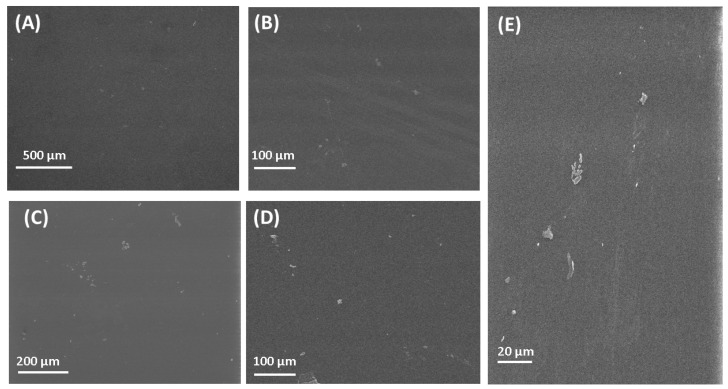
SEM images of polymers and composite materials. (**A**) PVA; (**B**) CS; (**C**) PVP; (**D**) PVA/PVP/CS; (**E**) PVA/PVP/CS/PDA.

The SEM images of the fabricated polymeric films can be seen in Figure 2. The SEM image of PVA is presented in Figure 2A. Figure 2A presents a clear image of PVA with its flat surface. The flat surface was also found for the PVA polymeric film by Jahan et al. [85]. The surface of chitosan films is also flat, which is in good agreement with research by [86]. The surface observed by SEM for other polymeric films fabricated in this research is flat, which is in good agreement with results published by other research groups [87,88,89,90]. SEM observation of the surface of polymeric films gave only general information, without the possibility to compare the surface roughness. For a detailed study of the surface, the AFM microscope was used. 

### 3.3. EDX Elemental Analysis

The elemental analysis results of polymers and composite materials have been given in Table 2.

**Figure 3:**
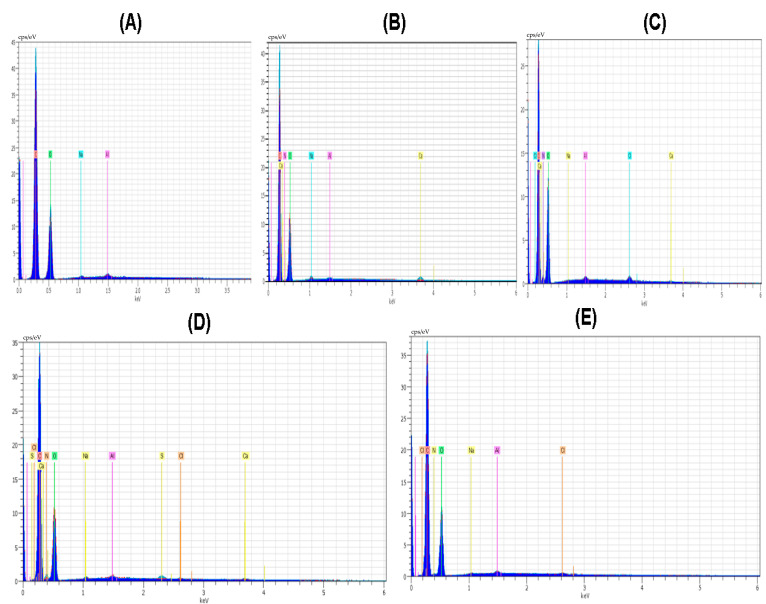
Spectrum of elemental analysis (**A**) PVA (**B**) PVP (**C**) CS (**D**) PVA/PVP/CS (**E**) PVA/PVP/CS/PDA.

Elements like C and O were noticeable in all polymer and composite material films. The amount of oxygen and nitrogen was found with the maximum value of 53.55 and 9.80 in the case of the CS polymeric film. Nitrogen was present in all the polymeric films except PVA; the main reason for this nitrogen absence is that the PVA structure did not involve nitrogen in the polymeric structure. The amount of nitrogen in the case of the PVP polymeric film was smaller; it was 4.81. The amount of oxygen in the case of polymeric film of PVA/PVP/CS was 48.84. The element Cl was present in the polymeric films except for PVA and PVP. The amount of Cl was highest with the value of 0.39 in the case of the polymeric film CS, and it was also present in the polymeric films of PVA/PVP/CS/PDA and PVA/PVP/CS with the values of 0.21 and 0.09, respectively. Al was observed with a maximum value of 0.56 in the case of PVA polymeric film, and Al presented a minimal value of 0.23 in the case of the polymeric film of PVA/PVP/CS/PDA. The spectrum of the elemental analysis of the polymers and composite materials is shown in Figure 3. Ruppel and Liang [83] presented the elemental analysis of polydopamine, and the elements reported in their study were carbon, nitrogen, oxygen, and chlorine.

### 3.4. Atomic Force Microscopy (AFM)

The surface topography of polymeric films made of CS, PVA, PVP, PVA/PVP/CS, and PVA/PVP/CS/PDA has been analyzed. The surface roughness results are presented in Table 3 in the form of the root mean square roughness (Rq) and arithmetic roughness average of surface (Ra) for individual polymeric films and polymeric composites. In the case of individual polymeric films, the highest values of Rq and Ra were noticed for CS film, with the values of 3.92 nm and 3.02 nm, respectively. The lowest values of Rq and Ra were noticed for PVP, 2.34 nm and 0.95 nm, respectively. The PVA individual polymeric film presented the values of Rq and Ra with the values of 3.38 nm and 2.11 nm, respectively. The composite polymeric film of PVA/PVP/CS presented maximum values of Rq and Ra with values of 11.91 nm and 8.71 nm, respectively. Adding polydopamine to a PVA/PVP/CS composite reduced Rq and Ra values to 7.49 nm and 5.15 nm, respectively. The results of surface roughness analysis indicate higher surface roughness for the polymeric composites than for individual polymers. Surface roughness has a vital role in wound healing, as it has been well explained by Khan et al. [91].

**Figure 4:**
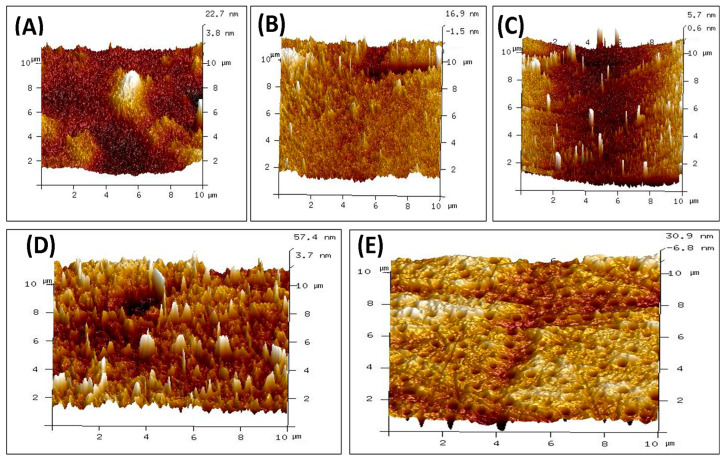
AFM images of polymers and composite materials (**A**) CS (**B**) PVA (**C**) PVP (**D**) PVA/PVP/CS (**E**) PVA/PVP/CS/PDA.

The AFM images of individual polymers and polymeric composites are presented in Figure 4. Surface roughness is crucial in many biomedical applications. Bhattacharjee et al. [92] explained the role of surface properties well, and it has been mentioned that tailoring the surface properties helps to achieve biocompatibility in biomaterials. Mao et al. highlighted that the surface roughness of biological materials plays a crucial role in affecting the polarization of macrophages. It has also been mentioned that surface roughness impacts the adhesion and spreading of the macrophages to the surfaces [93]. Maver et al. reported surface roughness in nanometers for prepared wound dressings [94]. Li et al. expressed that enhanced surface roughness can support cell adhesion [95]. Majhy et al. indicated the role of surface roughness in cell adhesion and proliferation [96]. Guo et al. reported that rough surfaces led to promoting platelet adhesion [97]. Verma et al. indicated in their study about the polymeric blend of chitosan and polyethylene oxide with the surface roughness in the range of 21 nm to 47 nm, and the polymeric blend was recommended for the wound dressing [98]. Ibrahim et al. in their study indicated the surface roughness of 2.08 nm for the blend of polyvinyl alcohol and sodium alginate loaded with the selenium nanoparticles, and the authors claimed about it for the wound dressing applications [99]. Kathyayani et al. prepared the blend in various ratios of poly(AVGVP)/collagen (100/0, 80/20, 60/40, 40/60, 20/80, 0/100), and they reported the surface roughness of 35.5 nm, 26.8 nm, 13.9 nm, 11.9 nm, 10.8 nm, and 8.96 nm, respectively. They conducted the in vivo testing for wound healing, which promoted the rate of wound healing [100]. Shabeena et al. utilized poly (vinyl alcohol) (PVA) nanocomposite films reinforced with halloysite nano-tubes (HNT), and it has been functionalized with chitosan. They used glutaraldehyde as the crosslinker. The reported values of the Rq and Ra in their study were 8.42 nm and 5.45 nm, respectively, and an in vitro study was conducted to analyze the prepared composite. The composite resulted in enhanced cell proliferation, and it led to tissue regeneration [101]. Walczak et al. prepared a composite based on collagen and thymol in the amounts of 4; 1; 0.75; 0.5; and 0.25 mg per cm^2^ of collagen and reported values of Rq in their study were 202 nm, 109 nm, 88.5 nm, 90.8 nm, and 49.5 nm. Authors recommended their blend for wound healing applications [102]. 

### 3.5. Mechanical Properties

**Figure 5:**
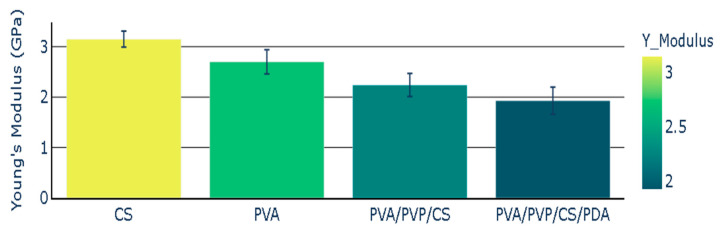
Young’s Modulus of polymers and polymeric composites.

The chitosan (CS) polymeric film presented Young’s modulus with a value of 3.15 GPa, while Young’s modulus value for PVA was 2.70 GPa. Young’s modulus for polymeric composite based on PVA, PVP, and CS presented the Young’s modulus with the value of 2.24 GPa. The addition of polydopamine (PDA) to polymeric composite (PVA/PVP/CS) led to a decrease in Young’s modulus. The polymeric composite containing polydopamine presented the Young’s modulus value of 1.93 GPa. Young’s modulus of polymers and polymeric composites is illustrated in Figure 5, and the mean values of Young’s modulus are presented with the standard error representation.

**Figure 6:**
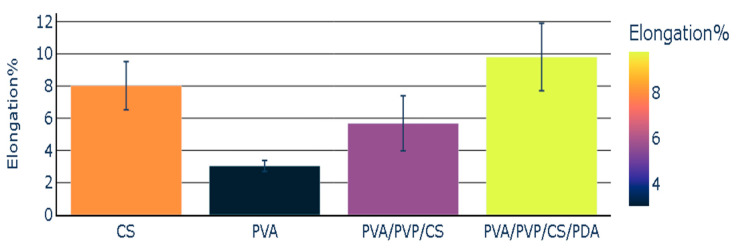
Elongation percentage of polymers and polymeric composites.

The elongation percentage was the lowest in the case of the individual polymeric film of PVA. CS polymeric film presented an elongation percentage with a value of 8.02. The elongation percentage noted in the case of polymeric composite (PVA/PVP/CS) was 5.68. This value was lower than the elongation percentage of the chitosan, but it was higher than the elongation percentage of polyvinyl alcohol. The polymeric composite (PVA/PVP/CS/PDA) containing polydopamine presented the highest value of the elongation percentage (9.80). PVA/PVP/CS/PDA presented the highest value of elongation percentage in comparison with the individual polymeric films and polymeric composite (PVA/PVP/CS), which did not contain polydopamine (PDA). The elongation percentage of polymers and polymeric composites has been reported in Figure 6, representing the mean values with the standard error values.

### 3.6. Contact Angle and Surface Energy Calculations 

Contact angle plays a notable role in analyzing the material’s contact with cells/tissues. In our research, the polymeric films were analyzed using glycerin and diiodomethane, and in both cases, the polymeric films presented a contact angle below 90°, which shows their hydrophilic nature. In the case of wound healing, the hydrophilic surface has a key role due to its tendency to hold the extra wound exudate. Kuo et al. highlighted the utilization of hydrophilic surfaces for the support of blood coagulation devices [103]. Wang et al. described that a moist environment is preferred for cell proliferation. Hydrophilic surfaces promote the release of growth factors, which is the prime reason for enhanced cell proliferation [104]. Xin et al. expressed that enhanced cell proliferation leads to early wound healing [105]. Dhania et al. also indicated the direct relationship between cell adhesion and wettability. It has also been expressed that cells prefer the hydrophilic surfaces to adhere rather than the hydrophobic surfaces [106]. The results of the contact angle and surface energy are presented in Table 4. IFT(s) represents the surface free energy, IFT(s, P) indicates the polar component of the surface free energy, and IFT(s, D) represents the dispersive component of the surface free energy. 

Chitosan polymeric film presented the surface free energy with a value of 36.01 mJ/m^2^, and surface free energy values for PVA, PVA/PVP/CS, and PVA/PVP/CS/PDA were 34.75 mJ/m^2^, 35.05 mJ/m^2^, and 31.29 mJ/m^2^. The surface free energy of studied films was the highest in the case of the polymeric film of chitosan, and it was smaller in the case of the polymeric film PVA/PVP/CS/PDA. The values for dispersive free energy for polymeric films of CS, PVA, PVA/PVP/CS, and PVA/PVP/CS/PDA were 33.13 mJ/m^2^, 30.90 mJ/m^2^, 31.78 mJ/m^2^, and 29.57 mJ/m^2^, respectively. The values of the polar component of the surface free energy for the polymeric films were 2.88 mJ/m^2^, 2.86 mJ/m^2^, 3.27 mJ/m^2^, and 1.72 mJ/m^2^. The role of surface-free energy in the cell-material interaction has been mentioned in various studies [107,108,109,110]. Metavarayuth et al. [107] indicated that surface-free energy has a role in the cell–biomaterials interactions, and it also has a key role in cell attachments. Michiardi et al. highlighted the role of surface-free energy in protein adsorption. Michiardi et al. have noted that the albumin adsorption has been increased with the increased polar surface free energy [111]. Comelles et al. [112] evaluated the role of surface-free energy in the case of polymeric materials like poly (methyl methacrylate), polystyrene, and poly (dimethylsiloxane) on serum protein adsorption. It has been concluded from their study that in the case of lower surface energy, the rate of the adsorption was found to be higher. Puzas et al.’s study expressed that the polar surface free energy impacts the polar molecules, and the polar molecules mentioned in the study were water and proteins. It has been stressed in their study that surface free energy may play a role in the development of biomedical devices [109]. Gentleman et al. described the decision criterion role of surface-free energy in the adsorbing species control and their bioactivity [113]. Cai et al. mentioned the role of surface-free energy in cell adhesion, and it has been highlighted in their study that higher surface-free energy is preferred for cell adhesion. It has also been indicated in their study that surface-free energy can be altered by the plasma treatment [114]. Surface free energy can be modified by polymer blending [108] and also by polymer crosslinking [115]. 

### 3.7. Cell Viability Evaluated Using the MTT Assay

The indirect evaluation of cytotoxicity of tested polymers (PVA and CS) and polymeric composites (PVA/PVP/CS and PVA/PVP/CS/PDA) was conducted using murine fibroblast-like cells L929 belonging to several well-suited cell lines recommended by the ISO standards as a model for cytotoxicity testing. The advantages of using these cells for this type of research include well-characterized and stable phenotypes, which ensure consistent and reproducible results across different experiments and laboratories, and sensitivity to a wide range of toxic substances, allowing for the detection of cytotoxic effects of various compounds [64,116]. Moreover, fibroblast-like cell lines serve as an appropriate model for investigating the biocompatibility of materials in wound healing and tissue engineering. It is well-established that fibroblasts play a crucial role in different phases of healing, such as crosstalk between fibroblasts and immune cells, production of extracellular matrix (ECM) components, and establishing crosstalk with endothelial cells and keratinocytes. Finally, fibroblasts are involved in remodeling the extracellular matrix by secreting matrix metalloproteinases (MMPs) and matrix components into a mature scar [117,118].

**Figure 7:**
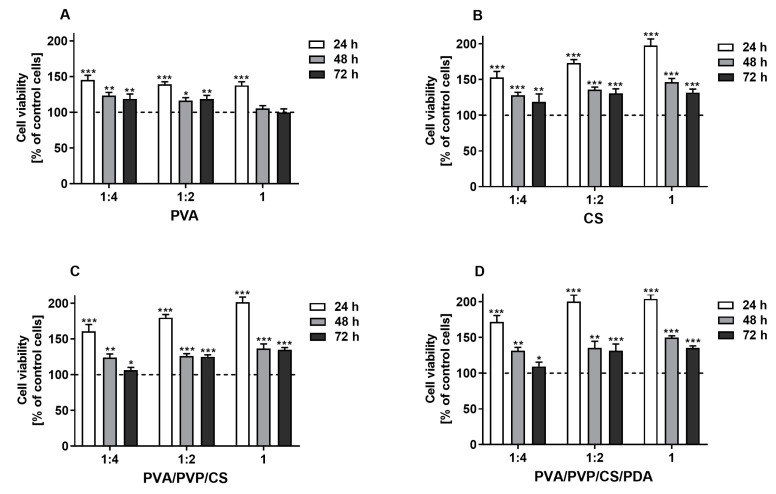
The viability of murine fibroblast cell line L929 stimulated with the extracts (undiluted (1) and diluted with culture medium 1:2 and 1:4) derived from tested polymers (PVA (**A**), CS (**B**)) and polymeric composites (PVA/PVP/CS (**C**), PVA/PVP/CS/PDA (**D**)) for 24, 48, and 72 h. Cell viability was presented as a percentage ± SDs of the control cells incubated in the corresponding dilution of culture medium, which was prepared similarly to the extracts. Asterisks show statistical differences between the control cells (served as 100%; dashed line) and the cells treated with the extracts (*** *p* < 0.001; ** *p* < 0.01; * *p* < 0.05).

The results of the MTT assay revealed that the tested extracts derived from polymers and polymeric composites promoted the proliferation of the L929 fibroblasts in a concentration-dependent manner after 24 h of cell stimulation, which was observed for all tested specimens, except for PVA polymer. Generally, compared with the control cells, the level of proliferation remained higher with extended incubation time, although the proliferation rate was lower than it was after 24 h. This phenomenon may be explained by the surface’s limiting area of wells within culture plates. Finally, the promotion of cell proliferation compared with control cells was not observed only with the undiluted and 1:2 diluted extracts derived from PVA films after 72 h of stimulation (Figure 7). Hence, it can be concluded that the prepared polymers and polymeric composites are non-toxic for L929 cells. However, additional studies are required to investigate the biocompatibility of tested samples and their potential utilization for biomedical applications. These findings are in line with studies previously published by other authors, who also showed biocompatible properties of materials based on polyvinyl alcohol, chitosan [119], polyvinyl pyrrolidone [120], and polydopamine [121] towards L929 cells. The results of our research contribute significantly to the existing knowledge on this topic, demonstrating that the modified blends of tested polymers and polymeric composites are also promising for biomedical applications. These findings suggest that the enhancements made to the materials improve their potential utility in various medical fields, including tissue engineering, wound healing, and the development of medical devices.

## 4. Conclusions

In the presented research, new composite material based on blends of polyvinyl alcohol (PVA), polyvinyl pyrrolidone (PVP), chitosan (CS), and polydopamine (PDA) has been successfully prepared. It was found that polymeric composites presented improved surface roughness compared with the individual polymeric materials. The surface roughness for the composite materials was higher than that for the individual polymers. The surface roughness was the highest in the case of the polymeric composite (PVA/PVP/CS) with the value of (Rq = 11.91 ± 0.66 nm and Ra = 8.71 ± 0.43 nm) and the lowest in the case of the PVP polymeric film with the value (Rq = 2.34 ± 0.64 nm and Ra = 0.95 ± 0.21 nm).

Mechanical properties such as Young’s modulus and elongation percentage of polymeric films were modified by adding PDA. The elongation percentage was improved in the case of the composite containing polydopamine. It increased in the case of the polymeric composite (PVA/PVP/CS/PDA) with the addition of PDA from 5.68% to 9.80%.

Contact angle measurements showed that the composite materials are hydrophilic, which is crucial in the cell proliferation phase of wound healing. The contact angle values for the polymeric films made of CS, PVA, PVA/PVP/CS, and PVA/PVP/CS/PDA were 78.8, 77.3, 78.5, and 86.1, respectively, in the case of glycerin, and in the case of diiodomethane were 46.0, 48.9, 48.0, and 54.4, respectively. The surface free energy was the highest in the case of the individual polymeric film made of chitosan with the value of 36.01 mJ/m^2^, and surface energy was reduced in the case of composite material made of PVA/PVP/CS/PDA with the value of 31.29 mJ/m^2^.

The results of the biological study showed that the modified blends of tested polymers and polymeric composites are promising for biomedical applications. 

## Figures and Tables

**Table 1:** Composition of composite materials.

Composites	PVA	CS	PVP	PDA
PVA/PVP/CS	40%	50%	10%	-
PVA/PVP/CS/PDA	40%	50%	5%	5%

**Table 2:** Percentage elemental analysis of polymers and composite materials.

Elements	PVA	PVP	CS	PVA/PVP/CS	PVA/PVP/CS/PDA
C	47.83	44.08	35.53	43.34	42.82
N	0	4.81	9.80	6.91	7.52
O	50.88	49.89	53.55	48.84	48.90
Al	0.56	0.30	0.30	0.24	0.23
Na	0.74	0.70	0.28	0.31	0.33
S	0	0	0	0.10	0
Cl	0	0	0.39	0.09	0.21
Ca	0	0.22	0.15	0.18	0

**Table 3:** Polymers/polymeric composites surface roughness.

Polymers/Polymeric Composites	Rq (nm)	Ra (nm)
PVA	3.38 ± 0.67	2.11 ± 0.23
PVP	2.34 ± 0.64	0.95 ± 0.21
CS	3.92 ± 0.73	3.02 ± 0.57
PVA/PVP/CS	11.91 ± 0.66	8.71 ± 0.43
PVA/PVP/CS/PDA	7.49 ± 0.35	5.15 ± 0.34

**Table 4:** Contact angle and surface energy of polymers and polymeric composites.

Polymers/Polymeric Composites	θ (Glycerin)	θ (Diiodomethane)	IFT(s) (mJ/m^2^)	IFT(s, D) (mJ/m^2^)	IFT(s, P) (mJ/m^2^)
CS	78.8 ± 2.522	46.0 ± 3.670	36.01	33.13	2.88
PVA	77.3 ± 3.627	48.9 ± 2.793	34.75	30.90	2.86
PVA/PVP/CS	78.5 ± 1.588	48.0 ± 2.822	35.05	31.78	3.27
PVA/PVP/CS/PDA	86.1 ± 1.597	54.4 ± 2.093	31.29	29.57	1.72

## Data Availability

Data reported in the article; further queries can be directed to the corresponding authors.

## Abbreviations

The list of the abbreviations in the manuscript is given below:

PVA	Polyvinyl Alcohol
PVP	Polyvinyl Pyrrolidone
CS	Chitosan
PDA	Polydopamine
SDS	Safety Data Sheet
SEM	Scanning Electron Microscopy
FTIR	Fourier Transform Infrared Spectroscopy
AFM	Atomic Force Microscopy
ATR	Attenuated Total Reflectance
ECM	Extracellular Matrix
MPPs	Matrix Metalloproteinases
ISO	International Organization for Standardization
EDX	Energy Dispersive X-Ray Spectroscopy
